# The value of metagenomic next-generation sequencing with blood samples for the diagnosis of disseminated tuberculosis

**DOI:** 10.3389/fcimb.2024.1456119

**Published:** 2024-12-09

**Authors:** Jing Ma, Yongfang Jiang, Yan He, Huaying Zhou

**Affiliations:** ^1^ Department of Infectious Diseases, the Second Xiangya Hospital, Central South University, Changsha, Hunan, China; ^2^ FuRong Laboratory, Changsha, Hunan, China; ^3^ Clinical Medical Research Center for Viral Hepatitis in Hunan Province, Changsha, Hunan, China

**Keywords:** metagenomic next-generation sequencing, peripheral blood, disseminated tuberculosis, clinical diagnosis, diagnosis performance

## Abstract

**Objective:**

The aim of this study was to assess the clinical value of metagenomic next-generation sequencing (mNGS) of blood samples for the identification of disseminated tuberculosis (DTB).

**Methods:**

A total of 48 individuals suspected of DTB were enrolled. All patients underwent mNGS of peripheral blood and conventional microbiological tests. Patient characteristics were collected from their medical records.

**Results:**

A total of 28 patients were diagnosed with DTB, whereas 20 patients were confirmed as non-DTB cases. In the DTB groups, 19 (67.9%) contained TB sequences, with specific reads of TB ranging from 1 to 219. The TB sequence was more detectable by mNGS in male patients, those with elevated PCT levels, those who are HIV positive, and those with a decreased CD4 T-cell count. The HIV-positive group shows higher TB mNGS reads (*p* = 0.012) and TB mNGS sensitivity (*p* = 0.05). The sensitivity of TB mNGS in blood samples was 80% for HIV-infected patients and 44.4% for non-HIV-infected individuals (*p* = 0.05). The non-HIV group had a higher prevalence of miliary tuberculosis (*p* = 0.018), and extrapulmonary tuberculosis was more prevalent in the HIV-positive group.

**Conclusion:**

Our research has shown that the mNGS of blood samples has excellent sensitivity for the diagnosis of DTB. The TB sequence was more detectable by mNGS in patients with elevated PCT levels, those who are HIV positive, and those with a decreased CD4 T-cell count.

## Introduction

Tuberculosis (TB) is the leading cause of morbidity and mortality in the HIV and non-HIV immunocompromised populations. TB primarily targets the respiratory system but can disseminate to other organs in the body. Disseminated TB (DTB), also known as miliary TB (MTB), is characterized by the systemic spread of the disease through tiny millet-sized lesions (1–4 mm) that can rapidly develop in various organs. DTB has become increasingly diagnosed in adults due to the growing prevalence of immunocompromising conditions such as diabetes, malignancy, and HIV/AIDS infections, as well as the increased use of immunosuppressive medications (Abuabat et al., 2023; Shafer et al., 1991; Sharma et al., 2012, 2016; Hassanein and Elbadry, 2016).

Diagnosing DTB is challenging due to nonspecific symptoms, atypical presentations, difficulties getting patient samples, and false-negative findings, leading to a delay in the diagnosis and increased mortality (Sharma et al., 2012; Hassanein and Elbadry, 2016). DTB has received less research attention than other forms of TB, at least in part because mycobacterial blood cultures are unavailable in most high-burden settings and have limited diagnostic value because median time to positivity is longer than median time from admission to death in fatal cases of DTB.

Metagenomic next-generation sequencing (mNGS) has been used to identify specific infectious pathogens in a timely, unbiased, and hypothesis-free manner (Zhang et al., 2014; Pang et al., 2024; Wu et al., 2024; Li et al., 2023; Liu et al., 2023; Gao et al., 2024; Shi et al., 2021; Zhou et al., 2019). It also has the ability to recognize co-infecting microbes. Furthermore, research indicates that mNGS can detect hematologic dissemination using blood tests, which may obviate the need for invasive procedures (Pang et al., 2024; Wu et al., 2024; Zhou et al., 2019). In recent years, accumulating data showed that mNGS is effective for diagnosing TB in direct clinical samples with excellent accuracy and specificity (Li et al., 2023; Liu et al., 2023; Gao et al., 2024; Shi et al., 2021; Zhou et al., 2019). Nevertheless, no research assessed the diagnostic accuracy of mNGS in patients with DTB. In this study, we explored the diagnostic efficacy of mNGS in DTB patients using blood samples.

## Patients and methods

This retrospective analysis consecutively enrolled 48 suspected DTB patients admitted to the Second Xiangya Hospital, Central South University (Changsha, China) from 1 May 2021 to 31 May 2024. Patients were eligible for enrollment if they met all the following criteria: (1) consent to undergo the plasma mNGS examination and (2) suspected DTB. Patients were excluded if they met any of the following criteria: (1) incomplete medical record and (2) patients already receiving antituberculosis therapy. Suspected DTB patients were included if they met at least one of the following criteria: (1) miliary pattern on chest imaging and (2) suspected bloodstream infections and TB exposure history. The final clinical diagnostic criteria for DTB include the following: (1) identifying either a miliary pattern on chest imaging and a positive MTB in sputum (culture, acid-fast staining smears, Xpert, or NGS) or lung biopsy showing granulomas, (2) active TB clinical manifestations and positive plasma mNGS results for MTB, or (3) detecting caseating granulomas in a biopsy from any other organ, along with the presence of a miliary pattern on chest imaging or a positive sputum culture for MTB. In the absence of microbiological evidence, the attending physician may diagnose DTB infection clinically by relating the patient’s clinical manifestations and imaging results to exclude other diseases, together with the patient’s confirmed responsiveness to anti-TB treatment after 1 month of follow-up. This study was approved by the Research Ethics Committee of the Second Xiangya Hospital, and the need for written informed consent was waived owing to its retrospective design.

### Clinical data collection

Data were collected from the patients’ medical records. The following details at diagnosis were gathered: demographics, presenting symptoms, imaging, and histopathological data, as well as risk factors and mortality rates. Mycobacterial blood culture and TB polymerase chain reaction (PCR)-based assays of blood samples cannot be collected due to the limitations of routine methods. In cases of incomplete data, historical manual files were retrieved and examined.

### Detection method (mNGS)

Specimens were obtained from patients according to standard procedures. Briefly, 3 mL of blood was collected in sterile DNase-free tubes. Plasma was separated by centrifuging the blood at 1,600 × *g* at 4°C for 10 min within 8 h of collection. Using high-throughput sequencing technology, the DNA sequence of the microorganisms in the sample was obtained and compared with that of the microbial gene bank to identify pathogenic microorganisms. The detection process includes nucleic acid extraction, library construction, computer sequencing, bioinformatic analysis, and report interpretation (Liu et al., 2023). The detection process was based on the Illumina NextSeq550Dx (Illumina) sequencing system, and reference databases for classification were downloaded from NCBI. RefSeq contains 11,958 bacterial genomes or scaffolds, 1,714 fungi related to human infection, 7,373 whole-genome sequences of viral taxa, and 343 parasites associated with human diseases. The number of TB reads was defined as the number of unique reads for the standardized species. MTB was considered positive when the mapping read number (genus or species level) was in the top 20 in the bacteria list (Miao et al., 2018).

### Statistical analysis

Continuous data are deemed as nonparametric. Continuous variables were expressed as median and interquartile range (IQR), and the Shapiro–Wilk test (*p* < 0.05) was used for continuous variables that exhibited non-normal distribution. The categorized data were represented by case number (*n*) and percentage (%). The chi-square test (McNemar test) was employed for comparison. *p* < 0.05 was statistically significant without adjustment for multiple tests. All analyses were conducted with the IBM SPSS statistical software, version 26.0.

## Results

### Patient profiles

**Figure 1:**
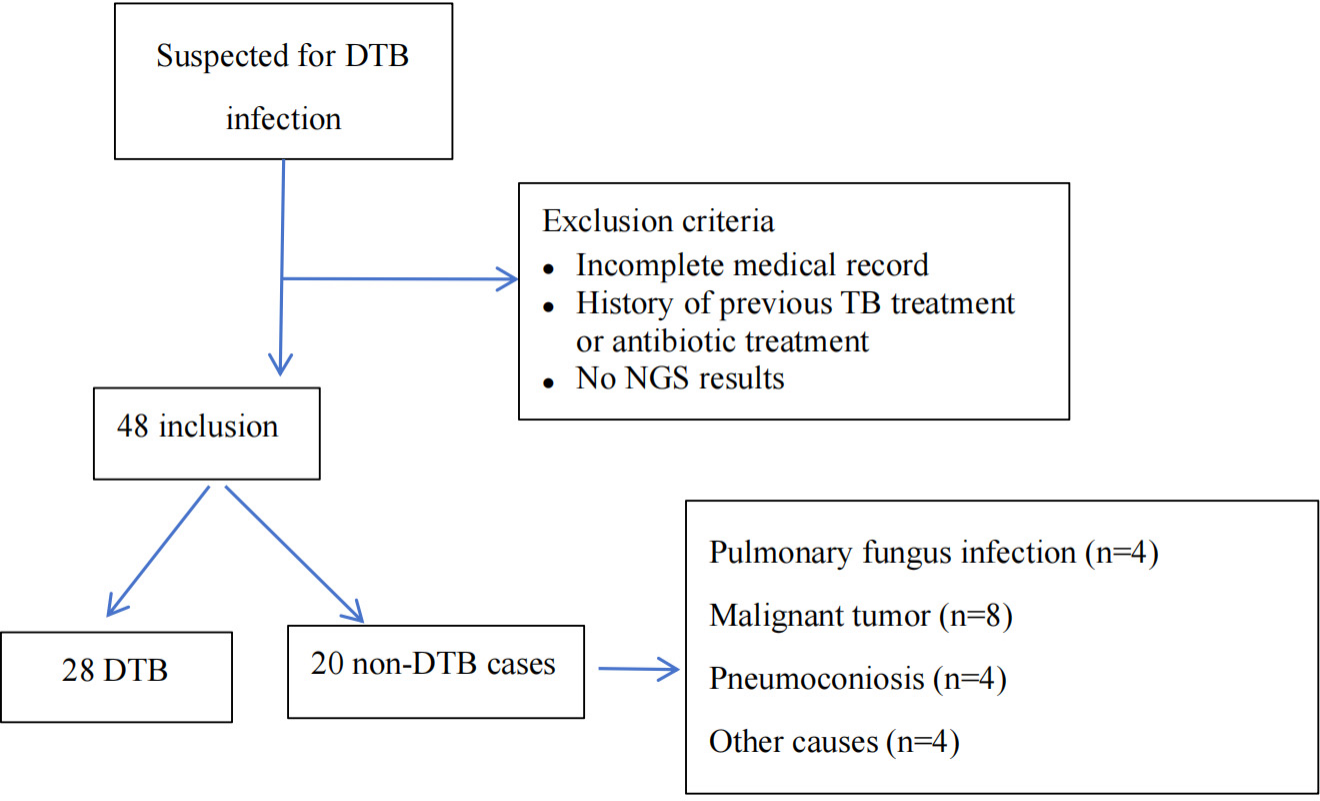
Flowchart of enrollment, exclusion criteria, and study procedures, by mNGS blood assay result. DTB, disseminated TB; NGS, next-generation sequencing.

Among 48 individuals with suspected DTB infection, 20 were identified as non-DTB cases (**Figure 1**). A total of 28 cases were confirmed DTB. **Table 1** summarizes the demographic characteristics of the 28 enrolled individuals diagnosed with DTB. The median age was 55 years, nearly 50% were over 60 years old, and 17 individuals (60.7%) were male. The predominant risk factor was HIV infection, present in 10 (35.7%) patients. Diabetes or pneumoconiosis was observed in four patients (14.3%); chronic liver disease was detected in 7.14% of patients; chronic kidney disease, anorexia, or pregnancy was observed in 3.6% of patients; and four (14.3%) patients had immunocompromising conditions, including malignancy or use of immunosuppressive agents. The most common presenting symptoms observed were fever, fatigue, and weight loss, occurring in 24 (85.7%), 20 (71.4%), and 23 (82.1%) patients, respectively. Cough was reported in 10 (35.7%) patients, night sweats in 14 (50%) patients, and hepatosplenomegaly in 16 (57.1%) patients. Regarding the anatomical site of TB infection at the time of diagnosis, the majority of patients had pulmonary MTB, which accounted for over half of the cases (16 patients; 57.1%). TB lymphadenitis and gastrointestinal involvement were the second most common sites, observed in six patients (21.4%), followed by pleural or peritoneal involvement in four patients(14.3%); and urogenital TB in one patient (3.6%).

**Table 1:** Demographic characteristics.

Characteristics [median (IQR) or *n* (%)]	*n* (%)
Age (years)	55 (38.75–64.75)
Elders (age ≥ 60 years)	13 (46.4%)
Male, *n* (%)	17 (60.7%)
Comorbidities	
HIV positive	10 (35.7%)
Chronic liver disease	2 (7.14%)
Diabetes	4 (14.3%)
Immunosuppression	4 (14.3%)
CKD	1 (3.6%)
Pneumoconiosis	4 (14.3%)
pregnancy	1 (3.6%)
Anorexia	1 (3.6%)
Symptoms	
Fever	24 (85.7%)
Cough	10 (35.7%)
Weight loss	23 (82.1%)
Night sweats	14 (50%)
Fatigue	20 (71.4%)
Hepatosplenomegaly	16 (57.1%)
TB type	
Miliary	16 (57.1%)
Lymphadenitis	6 (21.4%)
CNS	2 (7.14%)
Skeletal	2 (7.14%)
Gastrointestinal	6 (21.4%)
Pleural or Peritoneal	4 (14.3%)
Urogenital	1 (3.6%)
Hepatosplenic	2 (7.14%)

IQR, interquartile range; HIV, human immunodeficiency; CKD, chronic kidney disease; CNS, central nervous system.

**Table 2** outlines the most frequently observed laboratory findings in the study population. The most common laboratory abnormality was elevated ESR, present in 26 (92.9%) patients. Anemia was the second most prevalent laboratory finding, observed in 25 (89.3%) patients. Elevated liver enzymes were also frequently observed, with aspartate aminotransferase elevated in 12 (42.9%) patients, and alanine transaminase elevated in 11 (39.3%) patients. Leukopenia was present in 2 (7.14%) patients, thrombocytopenia in 4 (14.3%) patients, thrombocytosis or leukocytosis in 7 (25%) patients, and elevated creatinine in 7 (25%) patients. Positive T-SPOT.TB results were reported in 23 (82.1%) patients. CD4 T-cell subsets in the peripheral blood were analyzed in 22 individuals, of whom 16 (72.7%) showed a reduction. mNGS of blood samples was conducted on all patients; 19 (67.9%) contained TB sequences, with specific reads of TB ranging from 1 to 219. TB sequences were identified in 15 patients (53.6%) with over two reads and in 12 patients (42.9%) with over three reads. A total of 18 acid-fast bacilli (AFB) smears from sputum, pleural, or ascitic fluid were performed, of which 8 showed positive results. Bronchoalveolar lavage was performed five times, with two positive results. X-pert MTB/RIF was taken 14 times, with 6 positive outcomes. Positive biopsy results were observed in four patients with caseating granuloma.

**Table 2:** Laboratory findings.

	*N* = 28 (%)
Elevated ESR	26 (92.9%)
Anemia	25 (89.3%)
Leukopenia	2 (7.14%)
Thrombocytopenia	4 (14.3%)
Thrombocytosis	7 (25%)
Leukocytosis	7 (25%)
Elevated ALT	11 (39.3%)
Elevated AST	12 (42.9%)
Elevated creatinine	7 (25%)
Positive T-SPOT.TB results	23 (82.1%)
Decreased CD4 cells	16/22
TB reads by plasma mNGS > 2	15 (53.6%)
TB reads by plasma mNGS > 3	12 (42.9%)
Positive TB by plasma mNGS	19 (67.9%)
AFB smears taken	18
Positive AFB smears	8
BAL done	5
Positive BAL results	2
Xpert^®^ MTB/RIF taken	14
Positive Xpert^®^ MTB/RIF	6
Positive biopsy result	4

ESR, erythrocyte sedimentation rate; ALT, alanine aminotransferase; AST, aspartate aminotransferase; TB, tuberculosis; mNGS, metagenomic next-generation sequencing; AFB, acid-fast bacilli; BAL, bronchoalveolar lavage.

### Comparison of clinical features of DTB in patients with HIV infection and non-HIV infection

**Table 3** assesses the clinical characteristics of 18 DTB persons with non-HIV infections and 10 DTB patients with HIV infections. The HIV-positive group exhibits elevated TB mNGS readings (*p* = 0.012) and TB mNGS sensitivity (*p* = 0.05) compared to the non-HIV group. The sensitivity of TB mNGS in blood samples was 80% for HIV-infected patients and 44.4% for non-HIV-infected individuals (*p* = 0.05). The non-HIV cohort had a higher prevalence of MTB (*p* = 0.018), but extrapulmonary TB was more prevalent in the HIV-positive group. An older age was observed, although it lacked statistical significance in the non-HIV groups. No significant differences were seen between the two groups in regard to AST, ALT, WBC, HB, PLT, CRP, PCT, and Cr levels.

**Table 3:** Comparison of clinical features of DTB in patients with HIV infection and non-HIV infection.

Characteristics [median (IQR) or n (%)]	HIV Positve (10)	HIV Negative (18)	p
Age (years)	48.50 (28.50-61.50)	59.00 (40.25-72.25)	0.861
Elders (age>=60 years)	3 (30%)	10 (55.6%)	0.206
Male, n (%)	7 (70%)	10 (55.6%)	0.206
WBC	6.88 (5.80-9.85)	6.46 (5.37-13.90)	0.205
HB	93.00 (74.25-110.50)	85.00 (74.00-108.75)	0.886
PLT	257.00 (162.75-308.50)	203.00(124.25-326.25)	0.714
CRP	48.41(34.15-80.69)	75.43 (42.47-121.73)	0.968
ESR	68.50 (57.00-101.00)	53.00 (27.50-96.50)	0.896
PCT	0.51 (0.12-0.80)	0.75 (0.43-2.88)	**0.003**
ALT	31.60 (19.60-57.10)	24.10 (13.88-64.25)	0.769
AST	30.35 (26.58-52.60)	41.50 (26.05-61.98)	0.967
CD4	23.5(9.75-64.00)	218(121.00-408.00)	**0.012**
Military	2 (20%)	14 (82.3%)	**0.018**
TB mNGS sensitivity	8 (80.0%)	11 (44.4%)	**0.050**
Postive T-SPOT.TB results	9 (90.0%)	14 (82.3%)	0.080
TB reads by mNGS	3.00 (0.75-9.75)	1.00 (0-9.25)	**0.012**

DTB; disseminated tuberculosis; HIV, human immunodeficiency; IQR, interquartile range; WBC, white blood cell; HB, hemoglobin; PLT, platelets; CRP, C-reactive protein; ESR, erythrocyte sedimentation rate; PCT, procalcitonin; ALT, alanine aminotransferase; AST, aspartate aminotransferase; mNGS, metagenomic next-generation sequencing; TB, tuberculosis.

The bold values mean statistically significant values.

### Comparison of clinical features of DTB in mNGS-positive and -negative patients with TB

**Table 4** illustrates that mNGS of blood samples was performed in all patients; the diagnostic sensitivity of mNGS was calculated using the clinical composite diagnosis as reference standard. TB was identified by mNGS in 19 (67.9%) cases. The sensitivity of mNGS was 67.9%. In a comparison of TB mNGS-positive and -negative groups, TB sequences were more detectable by mNGS in male patients, those with elevated PCT levels, those who are HIV positive, and those with a decreased CD4 T-cell count. There were no significant differences between the two groups in terms of age, AST, ALT, WBC, HB, PLT, CRP, and Cr levels.

**Table 4:** Comparison of clinical features of DTB in mNGS-positive and mNGS-negative patients with TB.

Characteristics [median (IQR) or n (%)]	mNGS positive (19)	mNGS negative (9 )	p
Age (years)	55.00(46.00-60.00)	59.00(34.50-71.50)	0.908
Elders (age>=60 years)	8 (42.1%)	5 (55.5%)	0.739
Male, n (%)	14(73.7%)	3(33.3%)	**0.039**
WBC	6.75(4.36-12.41)	6.61(6.15-14.06)	0.555
HB	91.00(70.00-104.00)	86.00(75.50-110.50)	0.751
PLT	201.00(150.00-279.00)	303(154.50-463.50)	0.957
CRP	56.88(44.22-56.88)	44.09(18.8-131.91)	0.686
ESR	60.00(35.25-84.75)	88.00(37.5-100.00)	0.872
PCT	0.67(0.44-2.27)	0.63(0.12-2.05)	**0.021**
ALT	27.60(20.60-64.00)	22.60(9.70-51.00)	0.832
AST	33.30(26.80-64.00)	32.10(21.70-57.30)	0.422
Cr	69.50(53.00-91.50)	66.00(47.00-93.40)	0.973
Hepatosplenomegaly	11 (57.9%)	5 (55.6%)	0.491
Positive T-SPOT.TB results	15(78.9%)	8(88.9%)	0.220
CD4	71.00(17.75-160.25)	147.00(37-354.00)	**0.010**
Military	10 (52.6%)	6(66.6%)	0.491
HIV positive	8(42.1%)	2 (22.2%)	**0.009**

DTB, disseminated tuberculosis; TB, tuberculosis; mNGS, metagenomic next-generation sequencing; IQR, interquartile range; WBC, white blood cell; HB, hemoglobin; PLT, platelets; CRP, C-reactive protein; ESR, erythrocyte sedimentation rate; PCT, procalcitonin; ALT, alanine aminotransferase; AST, aspartate aminotransferase; Cr, creatinine; HIV, human immunodeficiency.

The bold values mean statistically significant values.

### Diagnostic performance of blood mNGS and T-SPOT.TB assay in confirmed DTB

**Figure 2:**
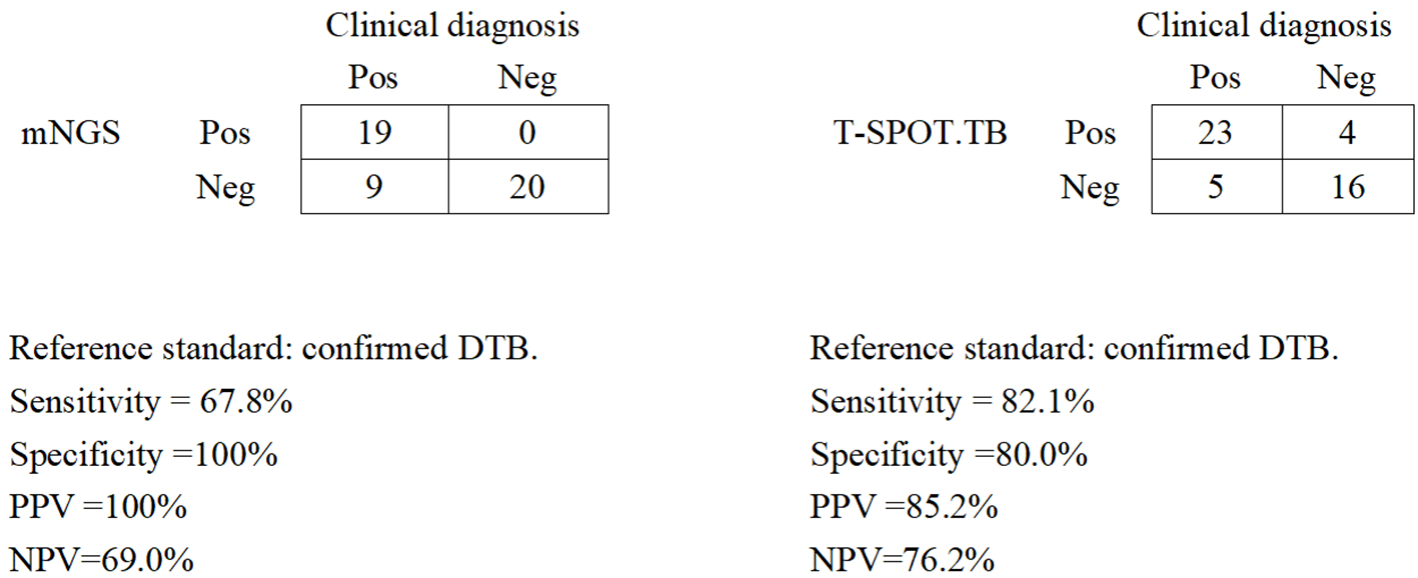
Diagnostic performance of mNGS and the T-SPOT.TB assay for DTB using blood samples. Pos, positive; Neg, negative; mNGS, metagenomic next-generation sequencing; DTB, disseminated tuberculosis; PPV, positive predictive value; NPV, negative predictive value.

We assessed the diagnostic performance for blood mNGS and T-SPOT.TB methods compared with the clinical final diagnosis of DTB (**Figure 2**). Of the 28 patients with confirmed DTB, 23 cases were detected by T-SPOT.TB, with a positive rate of 82.1%, while mNGS found 19 cases, with a positive rate of 67.8%. Among the 19 patients with positive mNGS, 4 patients had negative T-SOPT.TB assays, whereas the remaining 15 patients had both positive mNGS and T-SPOT.TB assay. In these 28 patients, the positive rates of mNGS and TSPOT were not (*p* > 0.05) statistically different. With clinically confirmed DTB as the reference standard, mNGS demonstrated a clinical sensitivity of 67.8%, a clinical specificity of 100%, a positive predictive value of 100%, and a negative predictive of 69.0%. With clinically verified DTB as the reference standard, the T-SPOT.TB assay had a clinical sensitivity of 82.1%, a clinical specificity of 80%, a positive predictive value of 85.2%, and a negative predictive value of 76.2%.

## Discussion

In light of the continuing HIV/AIDS epidemic and increasing use of immunosuppressive and cytotoxic drugs, the burden of DTB will continue to rise (Meira et al., 2019). DTB is a notoriously difficult disease to diagnose, with nonspecific symptoms and clinical abnormalities that are shared by many other infectious and noninfectious diseases. Mortality from this disease has remained high despite effective therapy being available. The early, timely, and correct diagnosis of DTB is a key factor for reducing mortality. This study applied mNGS of blood samples to evaluate its diagnostic efficacy for DTB and chose the use of clinical diagnosis rather than mycobacterial blood culture as a comparison because of the limitation of routine methods. Despite the lack of culture for clinical diagnosis, pathological findings and follow-up greatly reduced the possibility of misdiagnosis.

This study revealed HIV infection as the most prevalent risk factor for DTB, accounting for 35.7%; other risk factors were pharmacological immunosuppression, diabetics, chronic liver disease, and chronic kidney disease. Consistent with those reported in the literature (Ye et al., 2021; Crump et al., 2012; Kerkhoff et al., 2017), the importance of cell-mediated immunity was further illustrated by the inverse correlation found between the CD4+ T-cell count in HIV patients and the frequency of cases of DTB. Previous studies have highlighted the prognostic value of T-cell count in this context (Ye et al., 2021; Crump et al., 2012).

Several investigations from Africa with blood culture have shown that TB is a common cause of bloodstream infections (Crump et al., 2012; Kerkhoff et al., 2017). Blood is an attractive sample type for diagnosing DTB, especially for HIV-infected individuals, due to their higher probability of sputum culture-negative, disseminated, or extrapulmonary TB. Various studies on *Mycobacterium tuberculosis* bacteremia have established the potential of blood for TB detection by both culture and nucleic acid amplification test (NAAT) (Lima et al., 2009; Taci et al., 2003).

Mycobacterial blood culture is not available in most settings. Even where available, an average 3-week delay between culture and identification, combined with high early mortality, means that TB blood culture has limited diagnostic value. NAAT-based *M. tuberculosis* bacteremia studies have had different results, with sensitivities of TB detection in peripheral blood of 2% to 55% (Lima et al., 2009; Taci et al., 2003; Rebollo et al., 2006). Patients with DTB are at high risk for early death, and this sequential approach to empirical therapy introduces a potentially fatal delay in the commencement of anti-TB chemotherapy (ATC). Rapid testing of blood for TB with the mNGS may facilitate the rapid diagnosis of DTB and consequently the early initiation of ATC. Our findings revealed that 19 blood samples contained TB sequences from 28 diagnoses as DTB, indicating that plasma mNGS had a high sensitivity (67.9%) and that circulating cfDNA sequencing contributed to an improvement in the diagnosis of DTB. Our results demonstrated that TB sequence was more detectable by plasma mNGS in patients with HIV infection, elevated PCT levels, and decreased CD4 T-cell count. Patients who were HIV positive had higher TB mNGS reads and TB mNGS sensitivity. The sensitivity of TB mNGS in blood samples was 80% for HIV-infected patients and 44.4% for non-HIV-infected individuals. The data also revealed that immunocompromised patients were more likely to yield positive mNGS results, and detection of *M. tuberculosis* bacteremia by mNGS was rapid and had high specificity (100%) and high sensitivity (67.9%) compared to blood culture and NAAT.

In the diagnostic specificity of DTB, mNGS is better than T-SPOT.TB, although the T-SPOT.TB assay is slightly more sensitive than mNGS for DTB; a cost/time trade-off would be beneficial. In China, the mNGS test costs between $300 and $450 per sample, whereas the cost of the TSOPT.TB assay ranges from approximately $84 to $100. The detection period for mNGS is 1 day, while the detection time for TSPOT.TB assay takes approximately 48 h, and research indicates that mNGS can identify drug-resistant genes or mixed pathogens, altering the therapeutic management of TB patients and reducing the cost-effectiveness (Brown et al.,2015; Doyle et al.,2018; Votintseva et al., 2017).

mNGS is not recommended for routine use in mild/moderate infections due to its high cost (Chiu and Miller, 2019; Miller et al., 2018). Nonetheless, it is necessary to carry out adequate development in complex patients. MTB is an intracellular bacterium, and its DNA extraction is challenging, which may result in reduced susceptibility to mNGS. Moreover, the false positives of mNGS should be carefully monitored and assessed. MTB is not a common background bacteria in laboratory settings; the possibility for contamination of MTB in blood samples is lower than that in other sample types. Therefore, even when one specific read of a taxon is mapped to either the species or genus level, MTB infection should be considered. MTB was considered positive if it meets the following criteria: (1) its genus or species level is among the top 20 with the highest standardized specific reading number (SDSMRN); (2) it ranks first within its genus; and (3) its SDSMRN exceeds 1. To reduce the risk of contamination introduced before mNGS procedures, we made a strict sterile procedure during sample collection and a relative nucleic acid-free guideline during specimen preparation. In addition, two independent experienced clinics analyzed the mNGS results to see whether they were in accordance with the patient’s clinical manifestation and diagnosis.

There are some limitations to this study. First, the sample size included was relatively small, comprising only 28 cases. Thus, the sensitivity of diagnosis DTB for mNGS may not be accurately evaluated, *M. tuberculosis* bloodstream infection is underdiagnosed, false negatives will increase, and the negative predictive value could be overestimated. Prospective studies with a bigger sample size are needed to further evaluate the diagnostic value of mNGS in DTB. Second, the patients included did not undergo traditional culture; comparative analysis with conventional methods was limited by lack of information; therefore, prospective studies are needed to compare mNGS with traditional detection methods. Third, biases resulting from the selection and referral processes are potentially important, since these investigations were conducted in single centers. Despite some limitations, this investigation provides useful insights into the clinical value of plasma mNGS for identifying DTB. A comprehensive, multicenter study is needed to verify our findings.

In conclusion, our study showed that mNGS of blood samples exhibits exceptional sensitivity for diagnosing DTB. The TB sequence was more detectable by peripheral blood mNGS in patients who were HIV positive or with other immunosuppressive diseases. Blood mNGS should be conducted when diagnosing DTB is challenging, particularly in areas with a high prevalence of HIV presenting with severe sepsis in order to prevent the high early death rate. However, further refinement and larger-scale validation studies are necessary, especially in HIV-positive or other immunosuppressive populations.

## Data Availability

The raw data supporting the conclusions of this article will be made available by the authors, without undue reservation.
